# Altered cortical-striatal circuits connectivity is associated with psychotic symptoms in patients with first-episode, drug-naïve early-onset schizophrenia

**DOI:** 10.3389/fpsyt.2025.1695904

**Published:** 2026-01-05

**Authors:** Lu Wang, Ruishan Liu, Juan Liao, Fan Li, Lihua Zhuo, Hongwei Li

**Affiliations:** 1 Department of Radiology, The Third Hospital of Mianyang, Sichuan Mental Health Center, Mianyang, China; 2 Department of Psychiatry, The First Affiliated Hospital of Chongqing Medical University, Chongqing, China

**Keywords:** early-onset schizophrenia, functional connectivity, Granger causality analysis, resting-state functional magnetic resonance imaging, effective connectivity (EC)

## Abstract

**Background:**

Schizophrenia is recognized as a connectivity disorder. Although functional connectivity (FC) abnormalities are frequently reported in schizophrenia patients, findings remain inconsistent. Additionally, causal connectivity in early-onset schizophrenia (EOS) is underexplored, and the association between aberrant brain measures and psychotic symptoms remains unclear.

**Methods:**

Resting-state fMRI data were collected from 21 first-episode, drug-naïve EOS patients and 21 matched healthy controls (HCs). A voxel-wise meta-analysis was first used to identify the consistent brain regions with altered spontaneous functional activity in EOS. These regions served as seeds for subsequent FC analysis and Granger causality analysis (GCA), and the obtained functional brain measures were examined for their associations with psychotic symptoms.

**Results:**

Relative to HCs, EOS patients exhibited reduced FC between the left middle frontal gyrus (MFG) and right Cerebellum_8 as well as left Cerebellum_7b, while the connectivity between the right caudate nucleus (CAU) and right precuneus (PCUN) was increased. The increased FC between the right CAU and right PCUN was positively correlated with PSYRATS-delusion scores. Additionally, GCA revealed increased causal flow from the right CAU to right amygdala, while effective connectivity (EC) from the triangular part of the right inferior frontal gyrus to left MFG was inhibited, but no significant association was detected between these functional changes and psychotic symptoms.

**Conclusions:**

EOS not only showed aberrant FC in cortico-striato-cerebellar circuits, but also exhibited disrupted causal connectivity in striatal-amygdala circuits and within prefrontal cortex. Importantly, hyperconnectivity within the cortical-striatal circuits may represent a key neural mechanism underlying the psychotic symptoms of EOS.

## Introduction

Schizophrenia is a severe and highly disabling chronic progressive psychiatric disorder that affects approximately 24 million people worldwide (1). It is characterized by hallucinations, delusions, thought disorders, emotional dysregulation, and various degrees of cognitive impairment (2). Over the past few decades, despite extensive basic and clinical studies on schizophrenia conducted by numerous researchers, the exact etiology and pathogenesis of schizophrenia remain unclear to date. One of the main current views holds that the onset of schizophrenia may be related to long-term biological and neurological developmental disorders such as synaptogenesis, pruning, and myelination during adolescence and early adulthood, which may be caused by genetic factors, environmental factors, or the interaction of multiple factors such as genes and the environment (3). Early-onset schizophrenia (EOS) is an important subtype of schizophrenia defined as schizophrenia that onset before the age of 18 years. Compared to adult-onset schizophrenia, EOS typically presents with more severe symptoms, poorer adaptability and responsiveness to antipsychotic medications, and worse prognosis. However, EOS not only exhibits clinical and biological continuity with adult-onset schizophrenia but also shows higher genetic susceptibility and are less affected by potential confounding factors such as antipsychotic medications, environmental factors, and interaction with age-related neurodegeneration (4–6). Therefore, EOS samples offer a unique perspective for schizophrenia research. Research on patients with EOS may help reveal the underlying neurodevelopmental mechanisms of schizophrenia and enhance the understanding of its pathophysiological processes.

Resting-state functional magnetic resonance imaging (rs-fMRI) has been widely used in the study of neuropsychiatric disorders such as schizophrenia (7). Resting-state fMRI is typically used to investigate brain spontaneous neural activity at baseline, without requiring participants to perform specific tasks or receive external stimuli, thereby effectively avoiding the instability associated with task-based conditions (8). In recent years, several studies have utilized methods such as amplitude of low-frequency fluctuations (ALFF), fractional amplitude of low-frequency fluctuations (fALFF), and regional homogeneity (ReHo) to investigate changes in spontaneous brain functional activity in EOS patients (9–12). However, the findings of these studies have been inconsistent, particularly in terms of the localization of aberrant brain regions and the direction of functional activity changes. We previously conducted a meta-analysis using the Seed-based *d* Mapping with Permutation of Subject Images (SDM-PSI) method and found that EOS patients exhibited stable and consistent regional spontaneous brain activity alterations in the left middle frontal gyrus (MFG.L) and right caudate nucleus (CAU.R) during the resting state (13). These brain regions are not only involved in the regulation of decision-making and executive control (14), but may also be closely correlated with the positive psychotic symptoms of schizophrenia (15). Thus, it is necessary to comprehensively investigate the impact of the above-mentioned consistent vulnerable brain regions on whole-brain function, which may provide more valuable information for understanding the intrinsic neural mechanisms of schizophrenia.

It is well known that the different regions of the brain are not independent of each other, but rather work together as an interconnected system, with different brain regions interacting to perform various functions. Schizophrenia has long been recognized as a disorder characterized by “disconnection” of brain networks and circuits, with abnormal functional connectivity between brain regions potentially related to aberrant neurodevelopment (16–18). In order to characterize functional integration of the brain, functional connectivity (FC) analysis has been developed and widely used to study aberrant functional integration in multiple neuropsychiatric disorders (19). Previous evidence has revealed that the cortical-subcortical-cerebellar circuits play a critical role in the pathophysiology of schizophrenia (20, 21). Alterations in the core network of the cortical-subcortical-cerebellar circuit primarily involve the prefrontal cortex, parietal cortex, temporal cortex, striatum, and cerebellum (22–24). Notably, analogous to the well-described cortico-striato-thalamo-cortical circuits, the cortico-striato-cerebellar circuit can also be conceptualized as comprising parallel, functionally segregated subcircuits. These subcircuits are largely organized through distinct territories of the striatum: the associative striatum (primarily the caudate nucleus) is integral to cognitive processes and connects with prefrontal and parietal cortices; the sensorimotor striatum (primarily the putamen) is involved in motor control and loops with motor and somatosensory cortices; and the limbic striatum (primarily the nucleus accumbens/ventral striatum) subserves emotional and motivational functions through its connections with limbic structures like the amygdala and orbitofrontal cortex (25, 26). The cerebellum, in turn, is topographically connected with these cortical-striatal loops, forming closed-loop circuits that contribute to the integration and fine-tuning of motor, cognitive, and affective processes (27, 28). However, it remains unclear whether and how these alterations are associated with psychotic symptoms such as hallucinations and delusions. In addition, previous research findings have shown discrepancies in the regions and levels of abnormality across studies. For example, He et al. found that patients with schizophrenia exhibited increased resting-state functional connectivity between the bilateral cerebellum VI and multiple cortical/subcortical networks, such as the frontoparietal network (FPN), default mode network (DMN), and sensorimotor network (SMN) (29). Other studies have reported decreased connectivity within the frontal/parietal cortex-striatum-cerebellum network in schizophrenia patients (24). Zhuo et al. found that FC between right cerebellum VI and the prefrontal cortex and subcortical nuclei in patients with schizophrenia differed from that between the right cerebellum VI and the visual cortex and sensorimotor cortex (22). In summary, FC analysis provides a unique perspective for investigating the underlying neuropathological mechanisms of schizophrenia, but the results are inconsistent, which may be related to factors such as age of onset, duration of illness, and use of antipsychotic medications. Hence, in order to minimize the impact of these confounding factors, it is necessary to conduct FC analysis on first-episode drug-naïve EOS patients, which may help to more clearly reveal the potential neural mechanisms of this disease. Nevertheless, characterizing how one region or network influences another region or network is challenging for FC analysis. Granger Causality Analysis (GCA) is a promising method for characterizing effective connectivity (EC) which originated in the field of economics. It requires no prior knowledge and estimates Granger causality through vector autoregression models to determine whether the past value of one time series can correctly predict the current value of another time series (30). Previous studies based on GCA have revealed that facial working memory deficits in first-episode schizophrenia patients may be associated with reduced causal information flow into and out of the right middle frontal gyrus (MFG), right inferior frontal gyrus (IFG), right thalamus, and right postcentral gyrus (31). The cognitive impairment associated with first-episode schizophrenia may involve changes in causal connectivity between the prefrontal cortex and the striatum (32). Additionally, abnormal causal connectivity between key regions of the auditory, language, and memory networks, primarily involving the superior temporal gyrus, Wernicke’s area, Broca’s area, and hippocampus, may be related to the occurrence of auditory verbal hallucinations (AVH) in schizophrenia (33). While abnormal EC alterations involving multiple networks or regions have been identified in adult-onset schizophrenia patients, GCA has rarely been used to investigate the directionality or specificity of aberrant connectivity in first-episode, drug-naive EOS patients, and it remains unclear how the development of EC abnormalities contributes to the onset of EOS.

Therefore, based on the above factors, in the present study, we utilized data-driven ROIs based on the results of functional meta-analysis, which can more objectively explore their functional integration abnormalities with the entire brain compared to hypothesis-driven ROIs (34). On this basis, we investigated the functional and causal connectivity patterns between these ROIs and other brain regions in an independent dataset (21 first-episode drug-naïve EOS patients and 21 healthy controls). Subsequently, we further examined whether and how these brain measures were associated with clinical variables, particularly hallucinations and delusions symptoms assessed by the Psychotic Symptom Rating Scale (PSYRATS). Based on previous studies of adult schizophrenia (24, 35), we speculate that EOS patients may display aberrant functional integration in key regions of the cortico-striato-cerebellar circuit, which would be related to psychotic positive symptoms.

## Methods

### Study subjects

In the present study, A total of 26 patients with patients with EOS were consecutively recruited from the outpatient department of the Third Hospital of Mianyang/Sichuan Provincial Mental Health Center in China. Twenty-three age and gender matched healthy controls (HCs) were enrolled through advertisements at local high schools, and all control participants were volunteers who voluntarily participated in scientific research. All patients were independently assessed by two experienced psychiatrists based on the diagnostic criteria of the DSM-IV (SCID-CR). For inclusion in our study, patients had to fulfill the diagnostic criteria for schizophrenia in the Fourth Edition-Text Revision (DSM-IV-TR) and the following inclusion criteria: (1) age ranged from 13 to 18 years old; (2) patients with EOS onset between the ages of 13 and 18 years; (3) Illness duration less than 24 months; (4) no current or previous use of antipsychotic medication; (5) no comorbid Axis-I or Axis-II diagnosis. The exclusion criteria for all participants were as follows: (1) any history (past or present) of neurological disorders or a family history of inherited neurological conditions; (2) history of head trauma with loss of consciousness; (3) diagnosed alcohol or other substance abuse (current or historical); (4) claustrophobia; (5) severe organic disease; (6) and MRI contraindications. All patients were assessed for psychopathological symptoms using the Positive and Negative Syndrome Scale (PANSS) and Psychotic Rating Scales (PSYRATS). The fMRI data and clinical scales were collected after the definitive diagnosis and before the treatment.

The research protocol was approved by the Ethics Committee of the Third Hospital of Mianyang/Sichuan Provincial Mental Health Center and was conducted following the Declaration of Helsinki. All participants and their families voluntarily provided written informed consent prior to the start of the study after receiving detailed information about the research process.

### Data acquisition and preprocessing

Rs-fMRI images were acquired for each participant using a 3.0T MRI scanner (Magnetom Skyra, Siemens, Germany). The scanning procedure was conducted in the MRI room of the Mianyang Third Hospital/Sichuan Provincial Mental Health Center. The rs-fMRI scanning parameters used were identical to those employed in a previous study (36).

This study utilized the resting-state fMRI data processing assistant (DPARSF 4.2, Advanced Edition) within the MATLAB R2022b platform for fMRI data preprocessing (37), following the procedures described below: (1) converting the original DICOM-formatted images into the standardized Neuroimaging Informatics Technology Initiative (NIFTI) format; (2) remove the first 10 time points; (3) to reduce the impact of differences in slice acquisition times on the results, the remaining 245 consecutive volumes were corrected for slice time (using the middle slice as the reference layer); (4) in order to minimize the impact of head motion by the subjects, any subject with translation > 2 mm or rotation > 2° was excluded; (5) Spatial normalization was achieved using a two-part registration method, in which all exported images were segmented and registered to Montreal Neurological Institute (MNI) space (3 × 3 × 3 mm^3^); (6) Convolution smoothing was performed using an isotropic Gaussian smoothing kernel with a full width at half maximum (FWHM) of 6 mm to enhance the signal-to-noise ratio of the images; (7) Multivariate linear regression was used to remove the effects of covariates such as head motion parameters in the Friston-24 model (38), white matter (WM) signals, and cerebrospinal fluid (CSF) signals on blood oxygen level-dependent (BOLD) signals; and (8) To exclude the effects of low-frequency drift and high-frequency noise (such as respiratory and heart rhythms), we performed temporal band-pass filtering (0.01-0.08 Hz) and linear trend removal.

### Identifying EOS-related consistent vulnerable brain regions from meta-analysis

To minimize the effects of antipsychotic medication and duration of illness, we re-screened the datasets included in previous studies (13), only including datasets with study subjects who were first-episode drug-naïve EOS patients. We then performed a secondary meta-analysis on the remaining datasets using the Seed-based *d* Mapping with Permutation of Subject Images (SDM-PSI) software (version 6.23, https://www.sdmproject.com/) (specific methodological details for this analysis are provided in the “Voxel-wise meta-analysis for functional differences” section of **Supplementary Material 1**).

### Resting-state FC analysis

In voxel-wise meta-analysis, regions with significantly abnormal regional spontaneous functional activity in first-episode drug-naïve EOS patients were defined as regions of interest (ROIs) for further FC analysis. The ROI-based functional connectivity analysis was performed using the RESTPLUS toolkit (39). Briefly, the time series of all voxels within the ROI were extracted and averaged. The average time series of voxels within the ROI was then compared with the time series of all other voxels in the whole brain using Pearson’s correlation calculations. Subsequently, the functional connectivity correlation coefficients were subjected to Fisher’s Z-transformation to approximate a normal distribution.

### Granger causality analysis

In the present study, the consistent vulnerable brain regions identified in the voxel-wise meta-analysis were selected as regions of interest (ROIs) for subsequent GCA analysis. Granger causality analysis was employed to investigate the effective connectivity (EC) between each ROI and all voxels within the entire brain. The voxel-wise, coefficient-based GCA was performed on the gray matter mask using the REST toolbox (40). In GCA, voxel-wise path coefficient analysis quantifies how activity in one brain region affects another, where the time series of the ROI is defined as time series x, and the time series of each voxel in the whole brain is defined as time series y. Positive value of the effect connectivity from x-to-y may indicate excitatory effect, while negative value may indicate inhibitory effect. The effect connectivity analysis from y to x was used to estimate the positive or negative feedback effect from other voxels in the whole brain to the ROI. Regression coefficients (β) are calculated based on the average ROI time series across the entire brain, generating β maps from x-to-y and y-to-x for each subject. Finally, to improve normality for subsequent statistical analysis, Fisher’s Z-transformation was applied to standardize the β coefficients (41, 42).

### Statistical analysis

Intergroup differences in demographic and clinical characteristics between EOS patients and HCs were examined using SPSS software (IBM SPSS Statistics for Windows, version 26.0). Chi-square tests were used for categorical variables, and independent samples t-tests were used for continuous variables. Statistical evaluation used the two-tail test, with the significance level set as *p*-value < 0.05.

Statistical analysis of resting-state FC data (Z maps) from the two groups was performed using SPM12 (statistical parametric mapping; http://www.fil.ion.ucl.ac.uk/spm) software, and a two-sample t-test was used to analyze intergroup differences to explore changes in global brain functional connectivity. The Gaussian random field (GRF) theory multiple comparison correction approach was utilized to adjust *p* values at the cluster level, with *p* < 0.05 defined as cluster-level significance and *p* < 0.001 as voxel-level significance. The GCA data from the two groups were processed using the REST toolbox (40), and a two-sample t-test was used to analyze the EC differences between EOS patients and HCs. The results were corrected using the GRF theory multiple comparison correction method, with single voxel level *p* < 0.001 and cluster level *p* < 0.05. To investigate the potential correlation between abnormal brain connectivity changes in EOS patients and clinical variables (disease duration, PANSS scores, and PSYRATS scores), this study extracted the mean z-values of FC and EC values in brain regions with intergroup differences and conducted the two-tailed Pearson’s correlation analyses with each characteristic. The Bonferroni correction was applied for multiple comparisons of Pearson’s correlation *p*-values, and the significance threshold for correlation was set at 0.05/24.

## Results

### Demographic and clinical characteristics

Seven subjects were excluded due to excessive head movement, including five EOS patients and two HCs five EOS patients. Finally, statistical analyses were performed on 21 EOS patients and 21 HCs. **Table 1** shows the demographic and clinical information of all participants. There were no significant statistical differences between the EOS group and the HC group in terms of gender distribution (*p* = 0.75), age (*p* = 0.62), and educational level (*p* = 0.48).

**Table 1:** Information on demographic and clinical characteristics of the subjects.

Items	EOS	HC	*t/χ2*	P-value
Sample size	21	21	–	–
Age, years (range)	16.62±1.36 (14–18)	16.76±1.08 (14–18)	-0.50	0.62
Gender (male/female)	9/12	8/13	0.09	0.75
Education, years	10.62±1.72	10.95±1.28	-0.71	0.48
Duration of illness, months	5.81±5.00	–	–	–
PANSS				
Total scores	90.48±20.87	–	–	–
Positive symptoms scores	23.67±6.91	–	–	–
Negative symptoms scores	25.00±8.11	–	–	–
General psychopathology scores	41.81±11.28	–	–	–
PSYRATS				
AH scores	17.71±16.28	–	–	–
Delusion scores	16.43±2.29	–	–	–

Values are presented as mean ± standard deviation.

EOS, early-onset schizophrenia; HC, healthy controls; PANSS, Positive and Negative Syndrome Scale; PSYRATS, Psychotic Rating Scales; AH, auditory hallucination.

### Consistent vulnerable brain regions in EOS identified from the meta-analysis

Through a systematic screening process, 10 eligible original studies (11 datasets) were ultimately identified (see **Supplementary Material**, **Supplementary Figure S1**). The overall meta-analysis sample included 429 first-episode drug-naive EOS patients (185 males and 244 females) and 336 healthy controls (152 males and 184 females) (detailed demographic and clinical characteristics of the samples are provided in **Supplementary Materials**, **Supplementary Tables S1**, **S2**). The consistent vulnerable brain regions identified through meta-analysis included the left middle frontal gyrus (MFG.L) and right caudate nucleus (CAU.R), both of which displayed increased regional spontaneous functional activity (see **Supplementary Material**, **Supplementary Tables S3**, **Supplementary Figure S2**). These two clusters were selected as ROIs and saved as binary masks for subsequent resting-state FC and GCA analyses.

### Functional connectivity analysis

**Figure 1:**
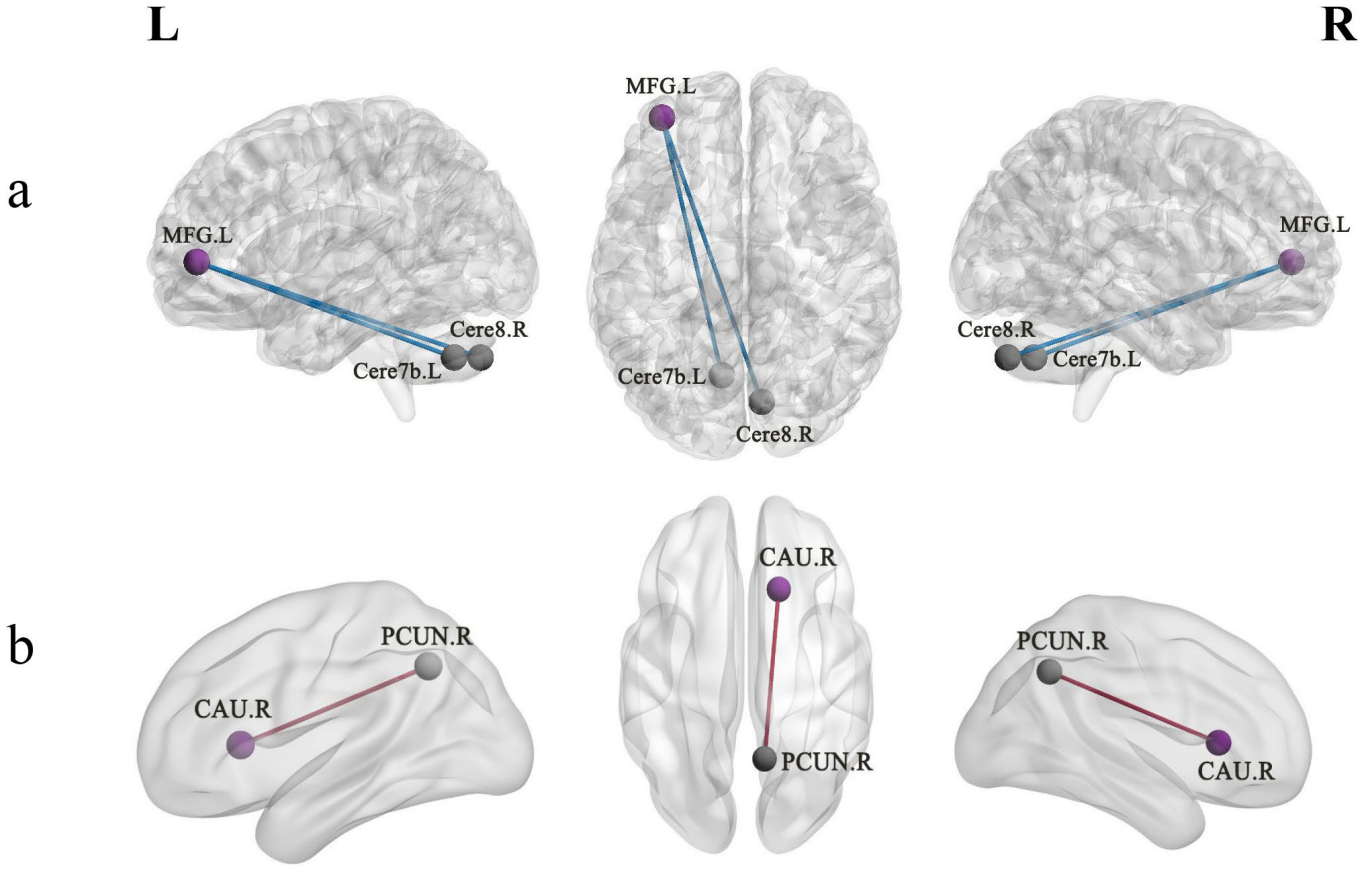
Altered FC between the ROIs (the left MFG and right CAU) and other brain regions in early-onset schizophrenia patients. **(a)** The 3D representation shows hypoconnectivity between the left MFG and the right Cerebellum_8 as well as left Cerebellum_7b (cold colors). **(b)** The 3D representation hyperconnectivity between the right CAU and right PCUN (warm colors). FC, functional connectivity; MFG, middle frontal gyrus; PCUN, precuneus; CAU, caudate nucleus; L, left; R, right.

When the left MFG was used as the ROI for FC analysis of resting-state fMRI data, we observed that the left MFG exhibited weaker resting-state FC with the right Cerebellum_8 and left Cerebellum_7b in EOS than in HCs (GRF corrected *p* < 0.05; **Table 2**; **Figure 1a**). In addition, when the right CAU was set as the ROI for analysis, compared with HCs, EOS patients showed increased connectivity between the right CAU and the right precuneus (PCUN) (GRF corrected *p* < 0.05; **Table 2**; **Figure 1b**).

**Table 2:** Difference in functional connectivity between the ROIs and other brain regions in EOS patients versus HCs.

Brain regions with significant FC alterations with the ROIs in EOS patients	Peak MNI coordinates			Cluster size (voxels)	Peak T value	*d*	95% CI for *d*		
	x	y	z				Lower	Upper	
Seed ROI: left MFG									
Right Cerebelum_8	9	-78	-39	157	-4.74	1.46	0.33		2.59
left Cerebelum_7b	-9	-66	-39	81	-4.52	1.39	0.26		2.52
Seed ROI: right CAU									
Right precuneus	9	-57	39	68	4.28	1.32	0.19		2.45

GRF correction was used to correct for multiple comparisons (voxel-level *p*<0.001, cluster-level *p*<0.05).

MNI, Montreal Neurological Institute; FC, functional connectivity; MFG, middle frontal gyrus; CAU, caudate nucleus; HCs, healthy controls; EOS, early-onset schizophrenia.

### Causal connectivity analysis

**Figure 2:**
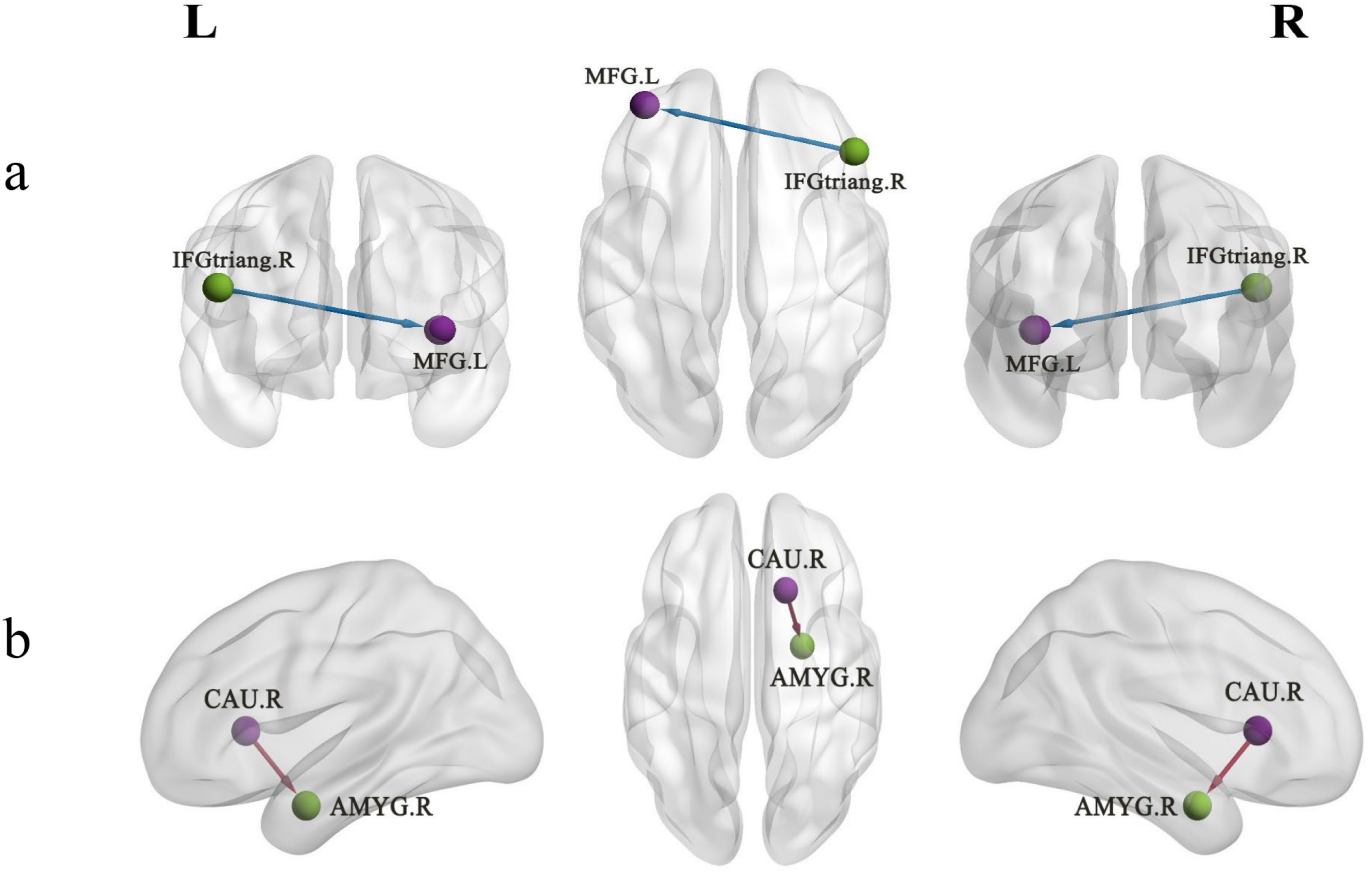
Altered EC between the ROIs (the left MFG and right CAU) and other brain regions in early-onset schizophrenia patients. **(a)** The 3D representation EC from the IFGtriang to the left MFG was inhibited (cold colors). **(b)** Increased causal flow from the right CAU to the right AMYG (warm colors). EC, effective connectivity; MFG, middle frontal gyrus; CAU, caudate nucleus; AMYG, amygdala; IFGtriang, the triangular part of the right inferior frontal gyrus; L, left; R, right.

The causal connectivity between the left MFG and other brain regions as well as between the right CAU and other brain regions was compared between the two groups of participants. The GCA results revealed that, compared with HCs, EOS patients showed EC from the triangular part of the right inferior frontal gyrus (IFGtriang) to the left MFG was inhibited in the resting state (*p* < 0.05, GRF corrected; **Table 3**; **Figure 2a**). In addition, we observed that in patients with EOS, compared with HCs, increased causal flow from the right CAU to the right amygdala (AMYG) (*p* < 0.05, GRF corrected; **Table 3**; **Figure 2b**).

**Table 3:** Aberrant effective connectivity in the patients with EOS compared with the HCs.

Brain region	Peak MNI coordinates					Cluster size (voxels)		Peak T value		*d*	95% CI for *d*		
	x	y		z							Lower	Upper	
x to y													
Right CAU to right amygdala	24	-3		-24		14		4.90		1.51	0.37	2.65
y to x													
From the right IFGtriang to left MFG	48	30		21		18		-5.25		1.62	0.47	2.77

GRF correction was used to correct for multiple comparisons (voxel-level *p*<0.001, cluster-level *p*<0.05).

MNI, Montreal Neurological Institute; EC, effective connectivity; CAU, caudate nucleus; MFG, middle frontal gyrus; IFGtriang, the triangular part of the right inferior frontal gyrus; HCs, healthy controls; EOS, early-onset schizophrenia.

### Correlation analysis

**Figure 3:**
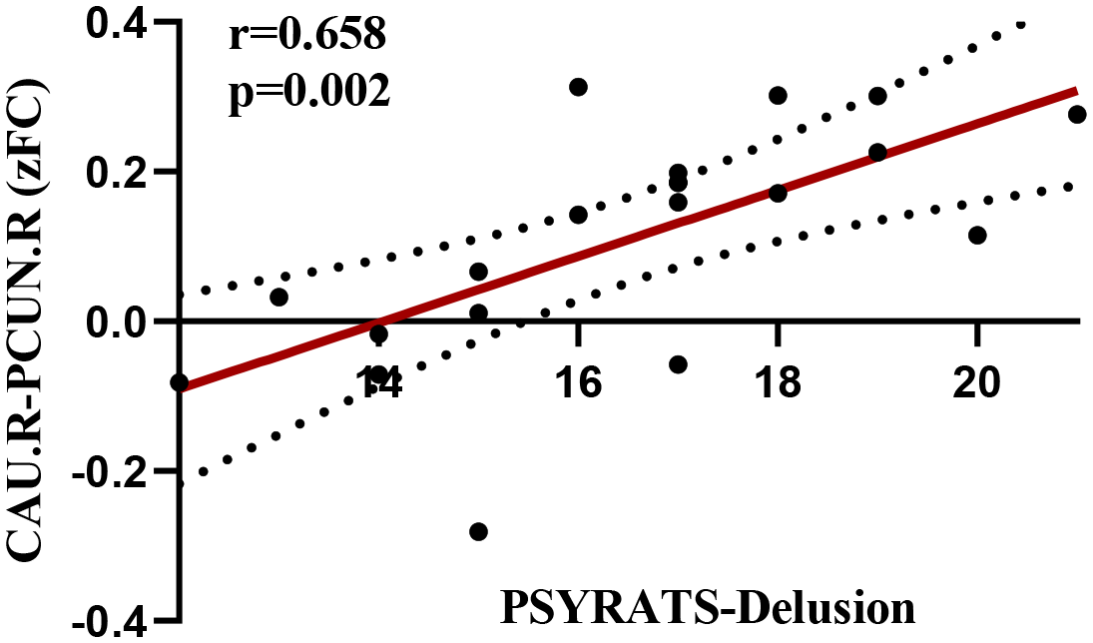
Correlation of FC between the right CAU and the right PCUN with the PSYRATS-delusion scores.

This study revealed that the altered FC between the right CAU and the right PCUN in patients with EOS was significantly positively correlated with PSYRATS-delusion scores (*r* = 0.658, *p* < 0.05/24), as shown in **Figure 3**. However, in patients with EOS, altered FC between the left MFG and right Cerebellum_8, between the left MFG and left Cerebellum_7b, as well as altered EC from the right IFGtriang to the left MFG and from the right CAU to the right amygdala, were not observed to correlate with PANSS-positive symptom scores (*r* = -0.040, *p* = 0.866; *r* = 0.011, *p* = 0.963; *r* = 0.117, *p* = 0.622; *r* = 0.031, *p* = 0.896), PANSS-negative symptom scores (*r* = 0.020, *p* = 0.932; *r* = 0.015, *p* = 0.949; *r* = -0.172, *p* = 0.470; *r* = 0.249, *p* = 0.289), PANSS-general psychopathology scores (*r* = -0.116, *p* = 0.636; *r* =- 0.068, *p* = 0.782; *r* = 0.045, *p* = 0.855; *r* = 0.021, *p* = 0.932), PANSS-total scores (*r* = -0.187, *p* = 0.444; *r* = -0.143, *p* = 0.558; *r* = 0.156, *p* = 0.524; *r* = 0.014, *p* = 0.954), PSYRATS-delusion scores (*r* = -0.140, *p* = 0.555; *r* = -0.158, *p* = 0.507; *r* = 0.301, *p* = 0.198; *r* = -0.137, *p* = 0.565), PSYRATS-auditory hallucination scores (*r* = 0.149, *p* = 0.532; *r* = 0.084, *p* = 0.725; *r* = -0.019, *p* = 0.938; *r* = 0.164, *p* = 0.489), or disease duration (*r* = -0.183, *p* = 0.440; *r* = -0.293, *p* = 0.210; *r* = 0.116, *p* = 0.628; *r* = 0.165, *p* = 0.486).

## Discussion

This study investigated the functional and causal connectivity changes in the entire brain of patients with first-episode drug-naïve EOS, and their correlations with clinical variables, based on data-driven ROIs. The main findings of the present study were that, when the left MFG was used as the ROI, EOS patients exhibited reduced functional connectivity between the left MFG and the right Cerebellum_8 as well as the left Cerebellum_7b, compared with HCs. When the right CAU was used as the ROI, increased connectivity was observed in the right PCUN, whereby the altered connectivity between the right CAU and right PCUN was significantly positively correlated with PSYRATS-delusion scores. These findings support the contribution of FC dysfunction in core brain regions of the cortico-striato-cerebellar circuits to the pathogenesis of EOS, and further suggest that hyperconnectivity between the DMN and striatum may play a critical role in the development of psychotic symptoms such as delusions in EOS patients. In addition, we used GCA resting-state fMRI data to respectively investigate the EC networks of two consistent vulnerable brain regions (the right CAU and left MFG) in EOS patients. The results showed increased driving effect from the right CAU to the right AMYG in EOS patients, while EC from the right IFGtriang to the left MFG was inhibited.

In the present study, reduced FC was observed between the left MFG and the right Cerebellum_8 as well as the left Cerebellum_7b in first-episode drug-naïve EOS patients. Cerebellar dysfunction has been suggested to may play a key role in the pathophysiological process of schizophrenia (43). The cognitive dysmetria hypothesis proposed by previous researchers suggests that aberrant FC between the cerebellum and the cerebral cortex may represent the most fundamental neurobiological change underlying various clinical symptoms in schizophrenia patients (44). The MFG is one of the key brain regions of the prefrontal cortex (PFC), playing a critical role in higher-order cognitive functions such as working memory, decision-making, and executive control (45). While the Cerebellum_8 and Cerebellum_7b regions are involved in important processes such as language processing, social cognition, and emotional regulation (46), abnormal FC between these regions may damage the cerebellum-prefrontal information integration circuit, leading to executive function deficits, impaired social interaction, and decreased emotional regulation capacity (47, 48). Brady et al. found that reduced resting-state FC between the cerebellum and the PFC was significantly correlated with negative symptoms in schizophrenia, and that repetitive transcranial magnetic stimulation (rTMS) can effectively modulate this FC abnormality, thereby improving patients’ negative symptoms (49). Evidence from a structural MRI study indicated that patients with persistent negative symptoms of schizophrenia exhibited smaller gray matter volume in the right PFC compared to patients with non-persistent negative symptoms (50). Additionally, another study revealed that the decoupling of FC between the cerebellum and PFC may also be underlying the expression of neurological soft signs (including sensory integration deficits and motor coordination disorders) in schizophrenia (20). These findings, together with the results of this study, support the disruption of cerebellum-prefrontal cortex connectivity in schizophrenia patients. During adolescence, long-range connectivity typically increases, while local functional connectivity decreases (51). The potential mechanism underlying these alterations in cerebellum-PFC connectivity may be the disruption of functional integration during early development, which may be associated with abnormalities in processes such as myelination, axonal terminal branching, and synapse formation (52). Hence, the results of this study still support the hypothesis of neurodevelopmental disorders in schizophrenia and further provide evidence for this hypothesis in terms of EOS. However, this study failed to observe any correlation between the functional disruption and schizophrenia negative symptoms. This inconsistency may be partly attributed to the clinical heterogeneity of the sample and the cross-sectional design, which makes it difficult to determine the exact timing of developmental changes.

In addition, we observed that, compared with HCs, FC between the right CAU and the right PCUN increased in EOS patients. The CAU, as a key node of the dorsal striatum, plays a crucial role in sensorimotor coordination, reward processing, and the processing and integration of cognitive information (53). The PCUN is the core hub of the DMN, and dysfunction in this region may lead to confusion in the perception of internal and external environments, abnormal self-referential processing, and impaired episodic memory and cognitive function (54, 55). Increased connectivity between the right CAU and the right PCUN suggested abnormal functional integration within the striatum-DMN loop in the early stages of schizophrenia. Previous studies have established the existence of the striatum-DMN loop (56, 57). A previous study showed that hyperconnectivity between the striatum and DMN in adults with schizophrenia (58). Disruption of the striatum-DMN loop has also been reported in adolescents with schizophrenia and high-risk individuals for schizophrenia (59, 60). Overall, these findings are consistent with the results of this study, suggesting that aberrant functional integration of the striatal-DMN loops may represent key pathophysiological changes in the early-stage of schizophrenia. In addition, the FC abnormalities observed above may also reflect specific impairments in different functional subregions within the caudate nucleus. According to the classical cortico-striato-thalamo-cortical circuits model, the caudate nucleus is not a functionally homogeneous structural node, but rather serves as a key functional hub integrating different functional networks (26). Specifically, the head of the caudate nucleus is primarily integrated into the executive control loop, connecting with regions such as the dorsolateral PFC to support higher-order cognitive functions (61). Whereas, the region of the caudate nucleus with abnormal connections to PCUN is more likely to correspond to the body of the caudate nucleus, which is integrated into the self-referential loop and works in concert with the DMN to participate in internally orientated thinking and the processing of self-referential information (62, 63). Therefore, the results of the present study further suggest that the caudate nucleus, as a functional hub, is specifically disrupted in the integration of different subregions within it with large-scale brain networks. However, it is worth noting that our findings are inconsistent with some previous studies, which reported that in schizophrenia patients, the breakdown of the striatal-DMN loops primarily manifested as overall hypoconnectivity (57). The inconsistent results may be attributed to various factors, including the disease stage, duration, and the use of antipsychotic medications during treatment. Therefore, we speculate that increased connectivity within the striatal-DMN circuits may be a characteristic of the early-stage of schizophrenia, while the observed decreased inter-regional FC may reflect disease progression.

Another interesting finding was that increased FC between the right CAU and right PCUN in EOS patients was significantly positively correlated with PSYRATS-Delusion scores, suggesting that hyperconnectivity between the striatum and DMN in schizophrenia patients may play a key role in the development of schizophrenia positive symptoms (mainly delusions) in the early stage of the disease. The striatum has long been considered to play an important role in the pathogenesis of positive symptoms (delusion, hallucination) in schizophrenia. Evidence from multiple studies indicated that dopamine synthesis and release in the striatum (especially the associative part) was significantly enhanced, which was associated with positive symptoms in schizophrenia (64, 65), and antipsychotic medications can effectively improve these symptoms by blocking dopamine D2 receptors (15). The PCUN is correlated with self-referential processing, which is also involved in the pathogenesis of delusion symptoms (66). For instance, Siemerkus et al. reported that increased functional activity in the right PCUN during virtual maze navigation was positively correlated with the severity of psychotic symptoms in schizophrenia patients (67). A recent study also found that FC between the PCUN and striatum was associated with the severity of delusions in schizophrenia (68). Therefore, both the striatum and PCUN have been reported to be related to the development of schizophrenia delusional symptoms, and the present study further supported this view by demonstrating hyperconnectivity between these two regions. The underlying mechanism for this change may be the abnormal coupling of the DMN, which is responsible for self-referential processing and internal thoughts, with the striatum-mediated salience attribution process, When irrelevant internal thoughts or sensory inputs are erroneously labeled by the striatum as highly salient (69).

In the present study, the GCA provided information on the EC direction between the right CAU and right AMYG. As a key node in the limbic system, the AMYG is primarily involved in the processing of threat signals, fear responses, and emotional valence (70). A previous study found that the sense of threat often accompanying delusions of persecution in schizophrenia patients may be related to increased activation of the AMYG (71). A recent study also reported that disruption of resting-state FC in the AMYG may be correlated with positive symptoms of schizophrenia (72). The anatomical connection between the striatum and the AMYG has been demonstrated by research, and the hyperactive signals of the striatal dopaminergic system may directly or indirectly overdrive the activity of the AMYG (73). Hence, enhanced EC between the two regions suggested excessive activity in the striatum-amygdala circuit, which may lead to emotional dysregulation, amplified threat perception, and the process of misattributing salience to psychotic stimuli that may provide a neural basis for positive symptoms such as delusions of schizophrenia (74, 75). Moreover, in EOS patients, causal flow from the right IFGtriang to the left MFG is reduced. The IFGtriang is a key region housing the motor language center, also known as Broca’s area, primarily involved in the processing of speech and language information (76). Damage to these functions appears to be frequently reported in schizophrenia (77, 78). Zhou et al. found that, compared with HCs, schizophrenia patients experienced inhibition of EC from the right MFG to the right IFG, which may be involved in the mechanism of facial working memory impairment in schizophrenia (31). However, no studies have yet investigated EC between the MFG and IFGtriang in EOS patients. Although aberrant functional and structural alterations in the MFG and IFGtriang were often mentioned in schizophrenia patients, the specific directionality of abnormal connectivity between these two regions remains unclear. In general, our findings may reflect the low efficiency of internal information transmission in the PFC in the early-stage schizophrenia, which holds significant implications for the pathogenesis of schizophrenia (32).

### Limitations

The present study has certain limitations. First, the strict recruitment criteria, including requirements for patients with first-episode and drug naïve, resulted in a very limited sample size, which may affect the statistical power and reliability of the findings. Overall, the present study is still a preliminary study, and multi-center collaborations are needed in the future to expand the sample size to further replicate and validate our findings. Second, this study had no restrictions on gender or clinical subtypes, making it impossible to determine the association between the pathogenesis of EOS and these factors. Therefore, further studies should be conducted based on different genders or clinical subtypes. Third, the samples recruited in this study and the participants included in our previous meta-analysis were all from the Chinese population, which may limit the generalizability of the current findings to other populations. Fourth, due to the limitations of the cross-sectional study design, we were unable to further explore the causal relationship between aberrant brain measurements and clinical variables in EOS patients. Longitudinal studies are needed to address this issue. Finally, it is worth noting that spatial heterogeneity in the efficiency of neural-vascular coupling may be confounded in the GCA results, resulting in a degree of reflection of vascular physiological properties rather than purely neural-driver relationships (79, 80). In addition, fMRI-based GCA analysis, as an exploratory method, have difficulty capturing millisecond neural interactions with its lower temporal resolution (81). These shortcomings might be compensated in future studies by multimodal data fusion of high temporal resolution electroencephalography (EEG)/magnetoencephalography (MEG) and fMRI, switching to dynamic causal modeling that quantifies changes in effective connectivity at the neuronal level, and the use of fMRI with high sampling rate (short repetition time) to optimize data acquisition.

## Conclusion

This study revealed FC dysfunction within the cortico-striato-cerebellar circuits in the early stage of schizophrenia, in which the hyperconnectivity of the cortical-striatal circuits may be an important neural basis for psychotic symptoms (the delusions). Additionally, abnormal causal connectivity patterns were observed in the striatal-amygdala circuit and within the PFC in EOS patients. These findings were independent of antipsychotic medication use. In summary, the results of this study support the dysconnectivity and neurodevelopmental disorder hypotheses of schizophrenia.

## Data Availability

The raw data supporting the conclusions of this article will be made available by the authors, without undue reservation. Requests to access these datasets should be directed to HL, lhw6321@163.com.

## References

[1] Abi-Dargham A . Schizophrenia: overview and dopamine dysfunction. J Clin Psychiatry. (2014) 75:e31. doi: 10.4088/JCP.13078tx2c, PMID: 25470107

[2] McCutcheon RA Reis Marques T Howes OD . Schizophrenia-an overview. JAMA Psychiatry. (2020) 77:201–10. doi: 10.1001/jamapsychiatry.2019.3360, PMID: 31664453

[3] Fatemi SH Folsom TD . The neurodevelopmental hypothesis of schizophrenia, revisited. Schizophr Bull. (2009) 35:528–48. doi: 10.1093/schbul/sbn187, PMID: 19223657 PMC2669580

[4] Hanssen M van der Werf M Verkaaik M Arts B Myin-Germeys I van Os J . Comparative study of clinical and neuropsychological characteristics between early-, late and very-late-onset schizophrenia-spectrum disorders. Am J Geriatr Psychiatry: Off J Am Assoc Geriatr Psychiatry. (2015) 23:852–62. doi: 10.1016/j.jagp.2014.10.007, PMID: 25500119

[5] Hu X Wang S Zhou H Li N Zhong C Luo W . Altered functional connectivity strength in distinct brain networks of children with early-onset schizophrenia. J Magnetic Resonance Imaging: JMRI. (2023) 58:1617–23. doi: 10.1002/jmri.28682, PMID: 36932678

[6] Wang X Liao W Han S Li J Wang Y Zhang Y . Frequency-specific altered global signal topography in drug-naïve first-episode patients with adolescent-onset schizophrenia. Brain Imaging Behav. (2021) 15:1876–85. doi: 10.1007/s11682-020-00381-9, PMID: 33188473

[7] Canario E Chen D Biswal B . A review of resting-state fMRI and its use to examine psychiatric disorders. Psychoradiology. (2021) 1:42–53. doi: 10.1093/psyrad/kkab003, PMID: 38665309 PMC10917160

[8] Yan H Xiao S Fu S Gong J Qi Z Chen G . Functional and structural brain abnormalities in substance use disorder: A multimodal meta-analysis of neuroimaging studies. Acta Psychiatrica Scand. (2023) 147:345–59. doi: 10.1111/acps.13539, PMID: 36807120

[9] Zheng J Zhang Y Guo X Duan X Zhang J Zhao J . Disrupted amplitude of low-frequency fluctuations in antipsychotic-naïve adolescents with early-onset schizophrenia. Psychiatry Res Neuroimaging. (2016) 249:20–6. doi: 10.1016/j.pscychresns.2015.11.006, PMID: 27000303

[10] Li YL Li YD Zhang H Gao ZT Xia YH Liang YH . Relationship between auditory hallucination and regional homogeneity of functional magnetic resonance imaging in first-episode childhood and adolescence-onset schizophrenia. Zhonghua yi xue za zhi. (2021) 101:1915–20. doi: 10.3760/cma.j.cn112137-20201126-03195, PMID: 34619853

[11] Wang S Zhang Y Lv L Wu R Fan X Zhao J . Abnormal regional homogeneity as a potential imaging biomarker for adolescent-onset schizophrenia: A resting-state fMRI study and support vector machine analysis. Schizophr Res. (2018) 192:179–84. doi: 10.1016/j.schres.2017.05.038, PMID: 28587813

[12] Jiang S Zhou B Liao Y Liu W Tan C Chen X . Primary study of resting state functional magnetic resonance imaging in early onset schizophrenia using ReHo. Zhong nan da xue xue bao Yi xue ban = J Cent South Univ Med Sci. (2010) 35:947–51. doi: 10.3969/j.issn.1672-7347.2010.09.008, PMID: 20871159

[13] Wang L Liu R Liao J Xiong X Xia L Wang W . Meta-analysis of structural and functional brain abnormalities in early-onset schizophrenia. Front Psychiatry. (2024) 15:1465758. doi: 10.3389/fpsyt.2024.1465758, PMID: 39247615 PMC11377232

[14] Cao W Li H Luo J . Prefrontal cortical circuits in social behaviors: an overview. J Zhejiang Univ Sci B. (2024) 25:941–55. doi: 10.1631/jzus.B2300743, PMID: 39626878 PMC11634449

[15] Agid O Mamo D Ginovart N Vitcu I Wilson AA Zipursky RB . Striatal vs extrastriatal dopamine D2 receptors in antipsychotic response–a double-blind PET study in schizophrenia. Neuropsychopharmacol: Off Publ Am Coll Neuropsychopharmacol. (2007) 32:1209–15. doi: 10.1038/sj.npp.1301242, PMID: 17077809

[16] Pettersson-Yeo W Allen P Benetti S McGuire P Mechelli A . Dysconnectivity in schizophrenia: where are we now? Neurosci Biobehav Rev. (2011) 35:1110–24. doi: 10.1016/j.neubiorev.2010.11.004, PMID: 21115039

[17] Kochunov P Hong LE . Neurodevelopmental and neurodegenerative models of schizophrenia: white matter at the center stage. Schizophr Bull. (2014) 40:721–8. doi: 10.1093/schbul/sbu070, PMID: 24870447 PMC4059450

[18] Friston K Brown HR Siemerkus J Stephan KE . The dysconnection hypothesis (2016). Schizophr Res. (2016) 176:83–94. doi: 10.1016/j.schres.2016.07.014, PMID: 27450778 PMC5147460

[19] Zhang J Kucyi A Raya J Nielsen AN Nomi JS Damoiseaux JS . What have we really learned from functional connectivity in clinical populations? NeuroImage. (2021) 242:118466. doi: 10.1016/j.neuroimage.2021.118466, PMID: 34389443

[20] Cai XL Wang YM Wang Y Zhou HY Huang J Wang Y . Neurological soft signs are associated with altered cerebellar-cerebral functional connectivity in schizophrenia. Schizophr Bull. (2021) 47:1452–62. doi: 10.1093/schbul/sbaa200, PMID: 33479738 PMC8379549

[21] Jensen KM King TZ Andrés-Camazón P Calhoun VD Iraji A . Aberrant cortical-subcortical-cerebellar connectivity in resting-state fMRI as an imaging marker of schizophrenia and psychosis: a systematic review of data-driven whole-brain functional connectivity analyses. Front Neuroimaging. (2025) 4:1650987. doi: 10.3389/fnimg.2025.1650987, PMID: 41140643 PMC12549315

[22] Zhuo C Wang C Wang L Guo X Xu Q Liu Y . Altered resting-state functional connectivity of the cerebellum in schizophrenia. Brain Imaging Behav. (2018) 12:383–9. doi: 10.1007/s11682-017-9704-0, PMID: 28293803 PMC5880870

[23] Grimaldi DA Patane A Cattarinussi G Sambataro F . Functional connectivity of the striatum in psychosis: Meta-analysis of functional magnetic resonance imaging studies and replication on an independent sample. Neurosci Biobehav Rev. (2025) 174:106179. doi: 10.1016/j.neubiorev.2025.106179, PMID: 40288705

[24] Cui LB Liu K Li C Wang LX Guo F Tian P . Putamen-related regional and network functional deficits in first-episode schizophrenia with auditory verbal hallucinations. Schizophr Res. (2016) 173:13–22. doi: 10.1016/j.schres.2016.02.039, PMID: 26995674

[25] Alexander GE DeLong MR Strick PL . Parallel organization of functionally segregated circuits linking basal ganglia and cortex. Annu Rev Neurosci. (1986) 9:357–81. doi: 10.1146/annurev.ne.09.030186.002041, PMID: 3085570

[26] Haber SN . Corticostriatal circuitry. Dialogues Clin Neurosci. (2016) 18:7–21. doi: 10.31887/DCNS.2016.18.1/shaber, PMID: 27069376 PMC4826773

[27] Buckner RL Krienen FM Castellanos A Diaz JC Yeo BT . The organization of the human cerebellum estimated by intrinsic functional connectivity. J Neurophysiol. (2011) 106:2322–45. doi: 10.1152/jn.00339.2011, PMID: 21795627 PMC3214121

[28] Bostan AC Strick PL . The basal ganglia and the cerebellum: nodes in an integrated network. Nat Rev Neurosci. (2018) 19:338–50. doi: 10.1038/s41583-018-0002-7, PMID: 29643480 PMC6503669

[29] He H Luo C Luo Y Duan M Yi Q Biswal BB . Reduction in gray matter of cerebellum in schizophrenia and its influence on static and dynamic connectivity. Hum Brain Mapping. (2019) 40:517–28. doi: 10.1002/hbm.24391, PMID: 30240503 PMC6865738

[30] Guo W Liu F Liu J Yu L Zhang J Zhang Z . Abnormal causal connectivity by structural deficits in first-episode, drug-naive schizophrenia at rest. Schizophr Bull. (2015) 41:57–65. doi: 10.1093/schbul/sbu126, PMID: 25170032 PMC4266300

[31] Zhou S Kuang Q Huang H She S Zheng Y Li X . Resting-state degree centrality and Granger causality analysis in relation to facial working memory in patients with first-episode schizophrenia. BMC Psychiatry. (2025) 25:147. doi: 10.1186/s12888-025-06535-7, PMID: 39972263 PMC11841165

[32] Huang H Shu C Chen J Zou J Chen C Wu S . Altered corticostriatal pathway in first-episode paranoid schizophrenia: Resting-state functional and causal connectivity analyses. Psychiatry Res Neuroimaging. (2018) 272:38–45. doi: 10.1016/j.pscychresns.2017.08.003, PMID: 29122402

[33] Gao J Zhang D Wang L Wang W Fan Y Tang M . Altered effective connectivity in schizophrenic patients with auditory verbal hallucinations: A resting-state fMRI study with granger causality analysis. Front Psychiatry. (2020) 11:575. doi: 10.3389/fpsyt.2020.00575, PMID: 32670108 PMC7327618

[34] Du Y Fu Z Calhoun VD . Classification and prediction of brain disorders using functional connectivity: promising but challenging. Front Neurosci. (2018) 12:525. doi: 10.3389/fnins.2018.00525, PMID: 30127711 PMC6088208

[35] Zeng LL Wang H Hu P Yang B Pu W Shen H . Multi-site diagnostic classification of schizophrenia using discriminant deep learning with functional connectivity MRI. EBioMedicine. (2018) 30:74–85. doi: 10.1016/j.ebiom.2018.03.017, PMID: 29622496 PMC5952341

[36] Zhou M Zhuo L Ji R Gao Y Yao H Feng R . Alterations in functional network centrality in first-episode drug-naïve adolescent-onset schizophrenia. Brain Imaging Behav. (2022) 16:316–23. doi: 10.1007/s11682-021-00505-9, PMID: 34410608

[37] Chao-Gan Y Yu-Feng Z . DPARSF: A MATLAB toolbox for “Pipeline” Data analysis of resting-state fMRI. Front Syst Neurosci. (2010) 4:13. doi: 10.3389/fnsys.2010.00013, PMID: 20577591 PMC2889691

[38] Friston KJ Williams S Howard R Frackowiak RS Turner R . Movement-related effects in fMRI time-series. Magnetic Resonance Med. (1996) 35:346–55. doi: 10.1002/mrm.1910350312, PMID: 8699946

[39] Jia XZ Wang J Sun HY Zhang H Liao W Wang Z . RESTplus: an improved toolkit for resting-state functional magnetic resonance imaging data processing. Sci Bull. (2019) 64:953–4. doi: 10.1016/j.scib.2019.05.008, PMID: 36659803

[40] Song XW Dong ZY Long XY Li SF Zuo XN Zhu CZ . REST: a toolkit for resting-state functional magnetic resonance imaging data processing. PloS One. (2011) 6:e25031. doi: 10.1371/journal.pone.0025031, PMID: 21949842 PMC3176805

[41] Hamilton JP Chen G Thomason ME Schwartz ME Gotlib IH . Investigating neural primacy in Major Depressive Disorder: multivariate Granger causality analysis of resting-state fMRI time-series data. Mol Psychiatry. (2011) 16:763–72. doi: 10.1038/mp.2010.46, PMID: 20479758 PMC2925061

[42] Zang ZX Yan CG Dong ZY Huang J Zang YF . Granger causality analysis implementation on MATLAB: a graphic user interface toolkit for fMRI data processing. J Neurosci Methods. (2012) 203:418–26. doi: 10.1016/j.jneumeth.2011.10.006, PMID: 22020117

[43] Cao H Argyelan M Yan J Velioglu HA Fang F Joanlanne A . Mapping cerebellar connectivity to cognition in psychosis: convergent evidence from functional magnetic resonance imaging and transcranial magnetic stimulation. Biol Psychiatry. (2025) 99:134–41. doi: 10.1016/j.biopsych.2025.06.023, PMID: 40617381 PMC12351477

[44] Andreasen NC Paradiso S O’Leary DS . Cognitive dysmetria” as an integrative theory of schizophrenia: a dysfunction in cortical-subcortical-cerebellar circuitry? Schizophr Bull. (1998) 24:203–18. doi: 10.1093/oxfordjournals.schbul.a033321, PMID: 9613621

[45] Nelson EE Guyer AE . The development of the ventral prefrontal cortex and social flexibility. Dev Cogn Neurosci. (2011) 1:233–45. doi: 10.1016/j.dcn.2011.01.002, PMID: 21804907 PMC3143481

[46] Schmahmann JD . The cerebellum and cognition. Neurosci Lett. (2019) 688:62–75. doi: 10.1016/j.neulet.2018.07.005, PMID: 29997061

[47] Cao H Wei X Zhang W Xiao Y Zeng J Sweeney JA . Cerebellar functional dysconnectivity in drug-naïve patients with first-episode schizophrenia. Schizophr Bull. (2023) 49:417–27. doi: 10.1093/schbul/sbac121, PMID: 36200880 PMC10016395

[48] Schmahmann JD . Dysmetria of thought: clinical consequences of cerebellar dysfunction on cognition and affect. Trends Cogn Sci. (1998) 2:362–71. doi: 10.1016/S1364-6613(98)01218-2, PMID: 21227233

[49] Brady RO Jr. Gonsalvez I Lee I Öngür D Seidman LJ Schmahmann JD . Cerebellar-prefrontal network connectivity and negative symptoms in schizophrenia. Am J Psychiatry. (2019) 176:512–20. doi: 10.1176/appi.ajp.2018.18040429, PMID: 30696271 PMC6760327

[50] Benoit A Bodnar M Malla AK Joober R Lepage M . The structural neural substrates of persistent negative symptoms in first-episode of non-affective psychosis: a voxel-based morphometry study. Front Psychiatry. (2012) 3:42. doi: 10.3389/fpsyt.2012.00042, PMID: 22586412 PMC3346965

[51] Dosenbach NU Nardos B Cohen AL Fair DA Power JD Church JA . Prediction of individual brain maturity using fMRI. Sci (New York NY). (2010) 329:1358–61. doi: 10.1126/science.1194144, PMID: 20829489 PMC3135376

[52] Fair DA Dosenbach NU Church JA Cohen AL Brahmbhatt S Miezin FM . Development of distinct control networks through segregation and integration. Proc Natl Acad Sci United States America. (2007) 104:13507–12. doi: 10.1073/pnas.0705843104, PMID: 17679691 PMC1940033

[53] Citro S Lazzaro GD Cimmino AT Giuffrè GM Marra C Calabresi P . A multiple hits hypothesis for memory dysfunction in Parkinson disease. Nat Rev Neurol. (2024) 20:50–61. doi: 10.1038/s41582-023-00905-z, PMID: 38052985

[54] Cavanna AE Trimble MR . The precuneus: a review of its functional anatomy and behavioural correlates. Brain: J Neurol. (2006) 129:564–83. doi: 10.1093/brain/awl004, PMID: 16399806

[55] Menon V . 20 years of the default mode network: A review and synthesis. Neuron. (2023) 111:2469–87. doi: 10.1016/j.neuron.2023.04.023, PMID: 37167968 PMC10524518

[56] Braskie MN Landau SM Wilcox CE Taylor SD O’Neil JP Baker SL . Correlations of striatal dopamine synthesis with default network deactivations during working memory in younger adults. Hum Brain Mapping. (2011) 32:947–61. doi: 10.1002/hbm.21081, PMID: 20578173 PMC3176660

[57] Wang X Li F Zheng H Wang W Zhang W Liu Z . Breakdown of the striatal-default mode network loop in schizophrenia. Schizophr Res. (2015) 168:366–72. doi: 10.1016/j.schres.2015.07.027, PMID: 26260079

[58] Salvador R Sarró S Gomar JJ Ortiz-Gil J Vila F Capdevila A . Overall brain connectivity maps show cortico-subcortical abnormalities in schizophrenia. Hum Brain Mapping. (2010) 31:2003–14. doi: 10.1002/hbm.20993, PMID: 20225222 PMC6870792

[59] Zhang Y Peng Y Song Y Zhou Y Zhang S Yang G . Abnormal functional connectivity of the striatum in first-episode drug-naive early-onset Schizophrenia. Brain Behav. (2022) 12:e2535. doi: 10.1002/brb3.2535, PMID: 35384392 PMC9120884

[60] Hua JPY Karcher NR Merrill AM O’Brien KJ Straub KT Trull TJ . Psychosis risk is associated with decreased resting-state functional connectivity between the striatum and the default mode network. Cognitive Affect Behav Neurosci. (2019) 19:998–1011. doi: 10.3758/s13415-019-00698-z, PMID: 30756347 PMC6690819

[61] Grahn JA Parkinson JA Owen AM . The cognitive functions of the caudate nucleus. Prog Neurobiol. (2008) 86:141–55. doi: 10.1016/j.pneurobio.2008.09.004, PMID: 18824075

[62] Zhang S Li CS . Functional connectivity mapping of the human precuneus by resting state fMRI. NeuroImage. (2012) 59:3548–62. doi: 10.1016/j.neuroimage.2011.11.023, PMID: 22116037 PMC3288461

[63] Harrison BJ Soriano-Mas C Pujol J Ortiz H López-Solà M Hernández-Ribas R . Altered corticostriatal functional connectivity in obsessive-compulsive disorder. Arch Gen Psychiatry. (2009) 66:1189–200. doi: 10.1001/archgenpsychiatry.2009.152, PMID: 19884607

[64] Kegeles LS Abi-Dargham A Frankle WG Gil R Cooper TB Slifstein M . Increased synaptic dopamine function in associative regions of the striatum in schizophrenia. Arch Gen Psychiatry. (2010) 67:231–9. doi: 10.1001/archgenpsychiatry.2010.10, PMID: 20194823

[65] Sorg C Manoliu A Neufang S Myers N Peters H Schwerthöffer D . Increased intrinsic brain activity in the striatum reflects symptom dimensions in schizophrenia. Schizophr Bull. (2013) 39:387–95. doi: 10.1093/schbul/sbr184, PMID: 22241165 PMC3576165

[66] Girard TA Lakatos L Menon M . Aberrant modulation of brain activation by emotional valence during self-referential processing among patients with delusions of reference. J Behav Ther Exp Psychiatry. (2017) 56:21–6. doi: 10.1016/j.jbtep.2016.11.007, PMID: 27887704

[67] Siemerkus J Irle E Schmidt-Samoa C Dechent P Weniger G . Egocentric spatial learning in schizophrenia investigated with functional magnetic resonance imaging. NeuroImage Clin. (2012) 1:153–63. doi: 10.1016/j.nicl.2012.10.004, PMID: 24179748 PMC3757729

[68] Miyata J Sasamoto A Ezaki T Isobe M Kochiyama T Masuda N . Associations of conservatism and jumping to conclusions biases with aberrant salience and default mode network. Psychiatry Clin Neurosci. (2024) 78:322–31. doi: 10.1111/pcn.13652, PMID: 38414202 PMC11488637

[69] Kapur S . Psychosis as a state of aberrant salience: a framework linking biology, phenomenology, and pharmacology in schizophrenia. Am J Psychiatry. (2003) 160:13–23. doi: 10.1176/appi.ajp.160.1.13, PMID: 12505794

[70] Andrewes DG Jenkins LM . The role of the amygdala and the ventromedial prefrontal cortex in emotional regulation: implications for post-traumatic stress disorder. Neuropsychol Rev. (2019) 29:220–43. doi: 10.1007/s11065-019-09398-4, PMID: 30877420

[71] Russell TA Reynaud E Kucharska-Pietura K Ecker C Benson PJ Zelaya F . Neural responses to dynamic expressions of fear in schizophrenia. Neuropsychologia. (2007) 45:107–23. doi: 10.1016/j.neuropsychologia.2006.04.026, PMID: 16814818

[72] Zhang M Yang F Fan F Wang Z Hong X Tan Y . Abnormal amygdala subregional-sensorimotor connectivity correlates with positive symptom in schizophrenia. NeuroImage Clin. (2020) 26:102218. doi: 10.1016/j.nicl.2020.102218, PMID: 32126520 PMC7052514

[73] Fudge JL Kunishio K Walsh P Richard C Haber SN . Amygdaloid projections to ventromedial striatal subterritories in the primate. Neuroscience. (2002) 110:257–75. doi: 10.1016/S0306-4522(01)00546-2, PMID: 11958868

[74] Aleman A Kahn RS . Strange feelings: do amygdala abnormalities dysregulate the emotional brain in schizophrenia? Prog Neurobiol. (2005) 77:283–98. doi: 10.1016/j.pneurobio.2005.11.005, PMID: 16352388

[75] Jalbrzikowski M Murty VP Tervo-Clemmens B Foran W Luna B . Age-associated deviations of amygdala functional connectivity in youths with psychosis spectrum disorders: relevance to psychotic symptoms. Am J Psychiatry. (2019) 176:196–207. doi: 10.1176/appi.ajp.2018.18040443, PMID: 30654642 PMC6420321

[76] Greenlee JD Oya H Kawasaki H Volkov IO Severson MA 3rd Howard MA 3rd . Functional connections within the human inferior frontal gyrus. J Comp Neurol. (2007) 503:550–9. doi: 10.1002/cne.21405, PMID: 17534935

[77] Hahn W Domahs F Straube B Kircher T Nagels A . Neural processing of nouns and verbs in spontaneous speech of patients with schizophrenia. Psychiatry Res Neuroimaging. (2021) 318:111395. doi: 10.1016/j.pscychresns.2021.111395, PMID: 34710797

[78] Pilon F Boisvert M Potvin S . Losing the chain of thought: A meta-analysis of functional neuroimaging studies using verbal tasks in schizophrenia. J Psychiatr Res. (2024) 169:238–46. doi: 10.1016/j.jpsychires.2023.11.013, PMID: 38048673

[79] David O Guillemain I Saillet S Reyt S Deransart C Segebarth C . Identifying neural drivers with functional MRI: an electrophysiological validation. PloS Biol. (2008) 6:2683–97. doi: 10.1371/journal.pbio.0060315, PMID: 19108604 PMC2605917

[80] Handwerker DA Ollinger JM D’Esposito M . Variation of BOLD hemodynamic responses across subjects and brain regions and their effects on statistical analyses. NeuroImage. (2004) 21:1639–51. doi: 10.1016/j.neuroimage.2003.11.029, PMID: 15050587

[81] Logothetis NK . What we can do and what we cannot do with fMRI. Nature. (2008) 453:869–78. doi: 10.1038/nature06976, PMID: 18548064

