# Supplementary material for: Altered cortical-striatal circuits connectivity is associated with psychotic symptoms in patients with first-episode, drug-naïve early-onset schizophrenia
Source: Front Psychiatry. 2026 Jan 5;16:1695904. doi: 10.3389/fpsyt.2025.1695904 (PMC12812569; doi:10.3389/fpsyt.2025.1695904)
Supplement: Supplementary file 1 [file DataSheet1.docx]

**Altered cortical-striatal circuits connectivity is associated with psychotic symptoms in patients with first-episode, drug-na****ïve early-onset schizophrenia**

**Content of supplementary materials**

Supplementary Methods: Identifying EOS-related consistent vulnerable brain regions from meta-analysis

Table S1. Demographic and clinical characteristics of included studies

Table S2. Imaging characteristics and quality scores of included studies

Table S3. Resting-state functional brain activity differences between first-episode drug-naïve EOS patients compared to HCs

Fig. S1. Flow diagram for the identification and exclusion of studies

Fig. S2. Voxel-wise meta-analysis results of difference between first-episode drug-naïve EOS and HCs

**Supplementary Methods: Identifying EOS-related consistent vulnerable brain regions from meta-analysis**

**Data source**

In the present study, we conducted a systematic and comprehensive search for studies on rs-fMRI in patients with first-episode drug-naïve EOS published in PubMed, Web of Science, Embase, Chinese National Knowledge Infrastructure (CNKI) and Wanfang databases through April, 2024, combined with the following keywords:(“schizophrenia” OR “schizophrenics” OR “schizophrenic disorder”) AND (“functional magnetic resonance imaging” OR “fMRI” OR “resting-state” OR “amplitude of low-frequency fluctuation” OR “ALFF” OR “fractional ALFF” OR “fALFF” OR “regional homogeneity” OR “ReHo”) AND (“adolescent” OR “child” OR “early-onset”). In addition, the references of the included studies and relevant review literature were examined to avoid the omission of other relevant studies.

**Study selection**

Studies were included in the meta-analysis if they satisfied the following criteria: (1) the study was original (rather than a review, meta-analysis, case report or abstract, etc.) that was peer-reviewed for publication in English or Chinese language journal；(2) the study subjects were formally diagnosed with schizophrenia before the age of 18 according to DSM, ICD or other criteria; (3) The study subjects were first-episode patients and had never received any antipsychotic medication treatment; (4) they analyzed resting-state functional brain activity at the whole-brain level; (5) they compared regional resting-state functional brain activity between patients with EOS and HCs；(6) peak coordinates based on whole-brain analysis were reported in three-dimensional stereotactic coordinates [Talairach or Montreal Neurological Institute (MNI)]；(7) If the study was a longitudinal or intervention trial, only baseline data were included for analysis. Exclusion criteria were：(1) patients with EOS were diagnosed with comorbid neurological or other psychiatric disorders; (2) they had fewer than 10 samples in a single group; (3) three-dimensional stereotactic coordinates of the peak of the activation point were unavailable, even after contacting the corresponding author by email or telephone; (4) the baseline data were unavailable; (5) the full-text could not be accessed; (6) If the data of different studies partially or completely overlapped, only the study with larger sample sizes and higher quality were included.

**Data extraction and quality assessment**

For each included study, we extracted the following information: (1) peak coordinates and effect values (e.g., *t*-values, etc.) of brain regions significantly different between patients with EOS and HCs; (2) the demographic and clinical characteristics, including sample size, gender, mean age, years of education, diagnostic criteria, medication status, duration of the illness, and PANSS scores; and (3) the imaging characteristics, including MRI scanner, method of analysis, data processing and analysis software used, the full width at half maximum (FWHM) parameter of the smoothing kernel, slice thickness and statistical thresholds used in brain imaging preprocessing.

**Voxel-wise meta-analysis for functional differences**

Meta-analysis of resting-state regional brain functional activity differences between individuals with first-episode drug-naïve EOS and HCs were conducted using the SDM-PSI software, respectively (version 6.23, https://www.sdmproject.com/). The SDM method has been described in detail in previous publications (1, 2). Briefly, we first extracted the peak coordinates and effect sizes (e.g., *t*-values) of brain regions with significant differences between patients with first-episode drug-naïve EOS and HCs reported in the original study. Then, a standardized MNI-based effect size signed map of functional differences between patients with PD and HCs was reconstructed for each dataset using an anisotropic non-normalized Gaussian kernel, respectively. Finally, these maps were consolidated into a standard random-effects model to obtain the mean maps, weighted by between-dataset heterogeneity, intra-dataset variability, and sample size, and pooled for multiple imputations using Rubin's rules. To optimally balance false positives and false negatives, the default Gaussian kernel size (FWHM = 20 mm) and threshold (peak height Z > 1, uncorrected *p* < 0.005, cluster extent > 10 voxels) were used, as recommended by the developer of SDM (3).

**Analyses of sensitivity, heterogeneity, and publication bias**

To evaluate the robustness and reproducibility of the main findings, a whole-brain voxel-based jackknife sensitivity analysis (i.e., iteratively repeating the same voxel-wise meta-analysis after excluding one different dataset each time) was performed. Between-study heterogeneity was quantitatively assessed using the *I^2^* statistic, with *I^2^* < 50% commonly indicating low heterogeneity (4). As for potential publication bias, funnel plots were created for visual inspection and quantitatively evaluated by Egger's tests. The Egger's test with *p*-value < 0.05 and asymmetric funnel plot were commonly considered significant for publication bias (5).

**Table S1 Demographic and clinical characteristics of included studies**

| **Study** | | **Subjects**  **(female)** | | | |  | | **Mean age/y** | | | |  | | **Education/y** | | | | **Diagnosis criteria** | | **Illness duration/m** | | **PANSS**  **scores** | | | | | | **Medication (%)** | | **First -episode** | |
| --- | --- | --- | --- | --- | --- | --- | --- | --- | --- | --- | --- | --- | --- | --- | --- | --- | --- | --- | --- | --- | --- | --- | --- | --- | --- | --- | --- | --- | --- | --- | --- |
|  |  | **EOS** | | **HCs** | |  | | **EOS** | | **HCs** | |  | | **EOS** | | **HCs** | |  | |  | | **Total** | | **Positive** | | **Negative** | |  | |  | |
| **Resting-state functional brain activity studies** | | | | | | | | | | | | | | | | | | | | | | | | | | | | | | | |
| Zheng  et al^(6)^. (2016) | | 35(15) | | 30(17) | |  | | 15.50 | | 15.43 | |  | | 8.70 | | 8.50 | | DSM-IV | | 6.60 | | 74.62 | | 20.42 | | 20.91 | | Drug-naive | | YES | |
|  | | | | | | | | | | | | | | | | | | | | | | | | | | | | | | | |
| Xiong  et al^(7)^. (2016) | | 20(12) | | 20(11) | |  | | 14.30 | | 14.40 | |  | | 7.30 | | 7.40 | | NA | | 8.90 | | 71.30 | | NA | | NA | | Drug-naive | | YES | |
|  | | | | | | | | | | | | | | | | | | | | | | | | | | | | | | | |
| Lü  et al^(8).^ (2016) | | 50(27) | | 33(18) | |  | | 14.20 | | 14.30 | |  | | 8.20 | | 8.30 | | DSM-IV | | 5.30 | | NA | | NA | | NA | | Drug-naive | | YES | |
|  | | | | | | | | | | | | | | | | | | | | | | | | | | | | | | | |
| Liang  et al^(9)^. (2018) | | 30(19) | | 30(16) | |  | | 13.00 | | 12.90 | |  | | NA | | NA | | DSM-IV | | 5.50 | | 74.50 | | NA | | NA | | Drug-naive | | YES | |
|  | | | | | | | | | | | | | | | | | | | | | | | | | | | | | | | |
| Pei  et al^(10)^. (2018) | | 33(16) | | 34(17) | |  | | 15.30 | | 15.60 | |  | | NA | | NA | | DSM-IV | | NA | | NA | | NA | | NA | | Drug-naive | | YES | |
|  | | | | | | | | | | | | | | | | | | | | | | | | | | | | | | | |
| Wang  et al^(11)^. (2018) | | 48(27) | | 31(17) | |  | | 15.79 | | 15.42 | |  | | 8.88 | | 8.44 | | DSM-IV | | 5..35 | | 75.10 | | 21.50 | | 17.92 | | Drug-naive | | YES | |
|  | | | | | | | | | | | | | | | | | | | | | | | | | | | | | | | |
| Li^a^  et al^(12)^. (2019) | | 26(21) | | 33(19) | |  | | 14.00 | | 13.58 | |  | | 6.46 | | 6.12 | | DSM-IV | | 2.00 | | 89.77 | | 26.42 | | 24.58 | | Drug-naive | | YES | |
|  | | | | | | | | | | | | | | | | | | | | | | | | | | | | | | | |
| Li^a^  et al^(12)^. (2019) | | 26(16) | | 33(19) | |  | | 13.96 | | 13.58 | |  | | 6.38 | | 6.12 | | DSM-IV | | 1.50 | | 93.65 | | 23.27 | | 24.81 | | Drug-naive | | YES | |
|  | | | | | | | | | | | | | | | | | | | | | | | | | | | | | | | |
| Lyu  et al^(13)^. (2021) | | 32(17) | | 27(17) | |  | | 16.75 | | 16.40 | |  | | 10.19 | | 10.11 | | DSM-IV | | 9.19 | | 79.44 | | 22.75 | | 16.97 | | Drug-naive | | YES | |
|  | | | | | | | | | | | | | | | | | | | | | | | | | | | | | | | |
| Li  et al^(14)^. (2021) | | 79(51) | | 32(18) | |  | | 14.10 | | 13.70 | |  | | 6.60 | | 6.80 | | DSM-IV | | 3.90 | | 90.20 | | 24.40 | | 22.90 | | Drug-naive | | YES | |
|  | | | | | | | | | | | | | | | | | | | | | | | | | | | | | | | |
| Liang  et al^(15)^. (2024) | | 50(23) | | 33(15) | |  | | 14.22 | | 14.33 | |  | | 8.22 | | 8.33 | | DSM-IV | | 5.26 | | NA | | NA | | NA | | Drug-naive | | YES | |

*Note*: ^a^The study included two subgroups, which we treated as two separate datasets.

*Abbreviations*: EOS, early-onset schizophrenia; HCs, healthy controls; y, year; m, month; NA, not available; PANSS, Positive and Negative Syndrome Scale; DSM, Diagnostic and Statistical Manual of Mental Disorders.

**Table S2 Imaging characteristics and quality scores of included studies**

| **Study** | **MRI scanner** | **Method of analysis** | | **Software** | | **Slice thickness**  **(mm)** | | **FWHM**  **(mm)** | | **Statistical threshold** | | **Quality scores** | |  |
| --- | --- | --- | --- | --- | --- | --- | --- | --- | --- | --- | --- | --- | --- | --- |
| **Resting-state functional brain activity studies** | | | | | | | | | | | | | |  |
| Zheng  et al^(6)^. (2016) | 3.0T | | ALFF | | DPARSF  SPM8  REST | | NA | | 6 | | AlphaSim(*p*_corrected_＜0.05) | | 9.5 | |
|  | | | | | | | | | | | | | | |
| Xiong  et al^(7)^. (2016) | 3.0T | | ALFF | | DPARSF  SPM8  REST | | 4 | | NA | | AlphaSim(*p*_corrected_＜0.05) | | 9.0 | |
|  | | | | | | | | | | | | | | |
| Lü  et al^(8)^. (2016) | 3.0T | | fALFF | | DPARSF SPM8  REST | | 4 | | 6 | | AlphaSim(*p*_corrected_＜0.05) | | 8.5 | |
|  | | | | | | | | | | | | | | |
| Liang  et al^(9)^. (2018) | 3.0T | | ALFF | | REST | | 1 | | NA | | AlphaSim(*p*_corrected_＜0.05) | | 9.0 | |
|  | | | | | | | | | | | | | | |
| Pei  et al^(10)^. (2018) | 3.0T | | ALFF | | REST | | 4 | | NA | | *p*_NA_＜0.01 | | 7.5 | |
|  | | | | | | | | | | | | | | |
| Wang  et al^(11)^. (2018) | 3.0T | | ReHo | | REST  DPARSF | | 4 | | 4 | | GRF(*p*_corrected_＜0.005) | | 10.0 | |
|  | | | | | | | | | | | | | | |
| Li  et al^(12)^. (2019) | 3.0T | | ALFF | | DPARSF  SPM  REST | | NA | | 6 | | AlphaSim(*p*_corrected_＜0.05) | | 9.0 | |
|  | | | | | | | | | | | | | | |
| Lyu  et al^(13)^. (2021) | 3.0T | | ReHo | | DPARSF  SPM12  REST | | 1 | | 6 | | AlphaSim(*p*_corrected_＜0.05) | | 9.5 | |
|  | | | | | | | | | | | | | | |
| Li  et al^(14)^. (2021) | 3.0T | | ReHo | | DPARSF  REST | | 4 | | 6 | | *p*_NA_＜0.05 | | 9.0 | |
|  | | | | | | | | | | | | | | |
| Liang  et al^(15)^. (2024) | 3.0T | | ALFF | | SPM8  REST | | 1 | | NA | | AlphaSim(*p*_corrected_＜0.05) | | 9.5 | |

*Abbreviations*: MRI, magnetic resonance imaging; FWHM, full width at half maximum; T, Tesla; ReHo, regional homogeneity; ALFF, amplitude of low-frequency fluctuations; fALFF, fractional amplitude of low-frequency fluctuations; NA, not available; DPARSF, data processing assistant for resting-state fMRI; REST, resting-state fMRI data analysis toolkit; SPM, statistical parametric mapping; GRF, Gaussian random fields; NA, not available.

**Table S3 Resting-state functional brain activity differences between first-episode drug-naive EOS patients compared to HCs**

| **Local Maximum** |  |  |  | **Cluster** | | **Egger’s test**  **(*p* value)** | **Heterogeneity (*I*^2^)** | **Jackknife**  **sensitivity analysis** |
| --- | --- | --- | --- | --- | --- | --- | --- | --- |
| **Region** | **Peak MNI coordinate**  **(x, y, z)** | **SDM-Z**  **value** | ***p* value** | **No. of voxels** | **Breakdown (No. of voxels)** |  |  |  |
| ***EOS vs. HCs (EOS＞HCs)*** | | | | | | | | |
| Right caudate nucleus | 16, 22, 6 | 3.62 | 1.46e^-5^ | 581 | Right anterior thalamic projections (272)  Right caudate nucleus (193)  Right caudate nucleus, BA 25 (45)  Corpus callosum (29)  (undefined) (41) | 0.98 | 23.45% | 9/11 |
|  | | | | | | | | |
| Left middle frontal gyrus, BA 10 | -36, 50, 4 | 3.82 | 6.75e^-5^ | 652 | Left inferior frontal gyrus, triangular part, BA 45 (139)  Left middle frontal gyrus, BA 46 (94)  Left anterior thalamic projections (72)  Left middle frontal gyrus, orbital part, BA 46 (72)  Left middle frontal gyrus, orbital part, BA 47 (64)  Left inferior frontal gyrus, orbital part, BA 47 (64)  Left middle frontal gyrus, BA 10 (45)  Left inferior frontal gyrus, orbital part, BA 46 (18)  Left inferior frontal gyrus, triangular part, BA 46 (17)  Left inferior network, inferior fronto-occipital fasciculus (17)  Left middle frontal gyrus, BA 47 (16) | 0.99 | 9.77% | 10/11 |

*Abbreviations*: HCs, healthy controls; EOS, early-onset schizophrenia; MNI, Montreal Neurological Institute; SDM, seed-based *d* mapping; BA, Brodmann area.

**Fig. S1 Flow diagram for the identification and exclusion of studies**
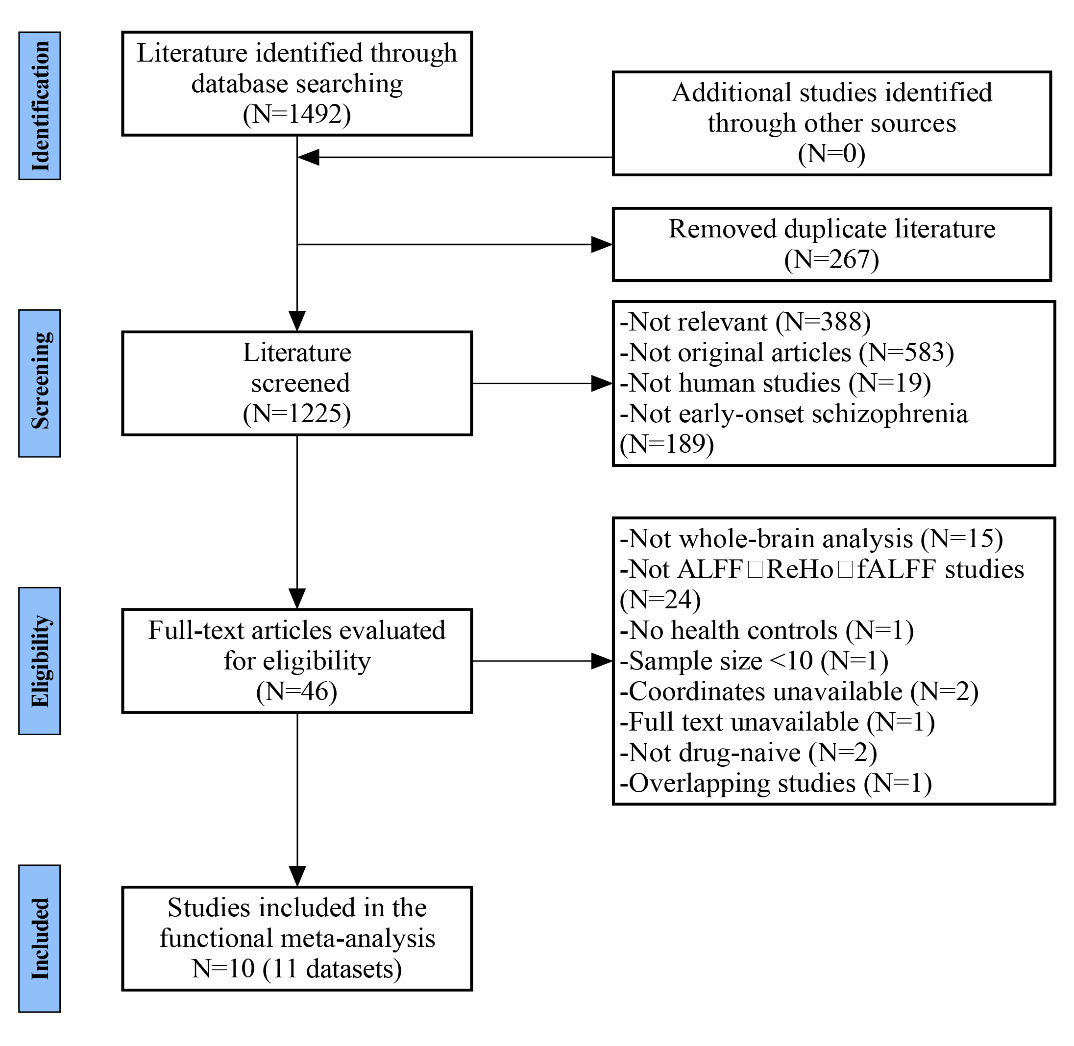


**Fig. S2 Voxel-wise meta-analysis results of difference between first-episode drug-naïve EOS and HCs
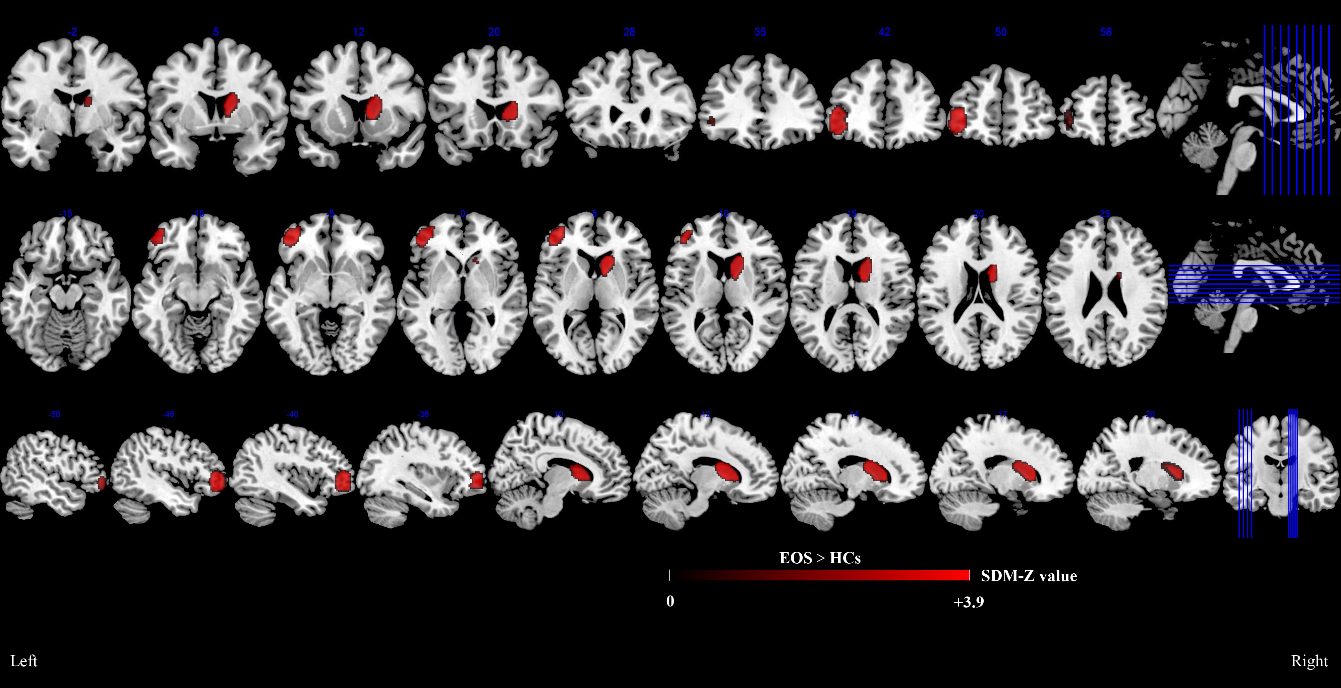
**

Regions with increased resting-state regional functional activity are displayed in red. The color bar indicates the maximum and minimum SDM-Z values. *Abbreviations*: EOS = early-onset schizophrenia; HCs = healthy controls; SDM = Seed-based *d* mapping.

**REFERENCES：**

1. Gao X, Zhang W, Yao L, Xiao Y, Liu L, Liu J, et al. Association between structural and functional brain alterations in drug-free patients with schizophrenia: a multimodal meta-analysis. Journal of psychiatry & neuroscience : JPN. 2018;43(2):131-42.

2. Radua J, Mataix-Cols D, Phillips ML, El-Hage W, Kronhaus DM, Cardoner N, et al. A new meta-analytic method for neuroimaging studies that combines reported peak coordinates and statistical parametric maps. European psychiatry : the journal of the Association of European Psychiatrists. 2012;27(8):605-11.

3. Radua J, Rubia K, Canales-Rodríguez EJ, Pomarol-Clotet E, Fusar-Poli P, Mataix-Cols D. Anisotropic kernels for coordinate-based meta-analyses of neuroimaging studies. Front Psychiatry. 2014;5:13.

4. Egger M, Davey Smith G, Schneider M, Minder C. Bias in meta-analysis detected by a simple, graphical test. BMJ (Clinical research ed). 1997;315(7109):629-34.

5. Sterne JA, Sutton AJ, Ioannidis JP, Terrin N, Jones DR, Lau J, et al. Recommendations for examining and interpreting funnel plot asymmetry in meta-analyses of randomised controlled trials. BMJ (Clinical research ed). 2011;343:d4002.

6. Zheng J, Zhang Y, Guo X, Duan X, Zhang J, Zhao J, et al. Disrupted amplitude of low-frequency fluctuations in antipsychotic-naïve adolescents with early-onset schizophrenia. Psychiatry research Neuroimaging. 2016;249:20-6.

7. Xiong YB, Ren Y, Cui XH, Xu WY, Sun XL, Yang H. Resting state fMRI study of amplitude of low-frequency fluctuation in early onset schizophrenia. Chin J Nerv Ment Dis. 2016;42(5):272-6.

8. Lü D, Shao RR, Liang YH, Xia YH, Guo SQ. [Fractional amplitude of low-frequency fluctuations in childhood and adolescence-onset schizophrenia: a resting state fMRI study]. Zhonghua yi xue za zhi. 2016;96(43):3479-84.

9. Liang Y, Shao R, Zhang Z, Li X, Zhou L, Guo S. Amplitude of low-frequency fluctuations in childhood-onset schizophrenia with or without obsessive-compulsive symptoms: a resting-state functional magnetic resonance imaging study. Archives of medical science : AMS. 2019;15(1):126-33.

10. Pei QL, Zhang HS, Wang B, Wang X, Zhao QJ. Study of Intracerebral Loop in Patients with Schizophrenia Without Early Onset of Schizophrenia by Resting State Functional Magnetic Resonance Imaging. Chin J CT MR. 2018;16(7):68-71.

11. Wang S, Zhang Y, Lv L, Wu R, Fan X, Zhao J, et al. Abnormal regional homogeneity as a potential imaging biomarker for adolescent-onset schizophrenia: A resting-state fMRI study and support vector machine analysis. Schizophrenia research. 2018;192:179-84.

12. Li YL, Li K, Wang B, Liang YH, Xia YH, Li YL, et al. Study of resting-state MRI in first-episode childhood and adolescence—onset schizophrenia with and without auditory hallucinatiolls. Chin J Nerv Ment Dis. 2019;45(8):454-9.

13. Lyu H, Jiao J, Feng G, Wang X, Sun B, Zhao Z, et al. Abnormal causal connectivity of left superior temporal gyrus in drug-naïve first- episode adolescent-onset schizophrenia: A resting-state fMRI study. Psychiatry research Neuroimaging. 2021;315:111330.

14. Li YL, Li YD, Zhang H, Gao ZT, Xia YH, Liang YH, et al. [Relationship between auditory hallucination and regional homogeneity of functional magnetic resonance imaging in first-episode childhood and adolescence-onset schizophrenia]. Zhonghua yi xue za zhi. 2021;101(24):1915-20.

15. Liang Y, Shao R, Xia Y, Li Y, Guo S. Investigating amplitude of low-frequency fluctuation and possible links with cognitive impairment in childhood and adolescence onset schizophrenia: a correlation study. Frontiers in psychiatry. 2024;15:1288955.
